# INTERSECTIONAL DIFFERENCES IN HEALTHY AGING WITH HIV

**DOI:** 10.1093/geroni/igz038.2810

**Published:** 2019-11-08

**Authors:** Tonya N Taylor

**Affiliations:** SUNY Downstate Medical Center, Brooklyn, New York, United States

## Abstract

Intersecting stigmatized social identities (i.e., old, Black, gay, HIV+) and structural forms of privilege and oppression (ageism, racism, homophobia, and HIV stigma) can contribute to poor psychological wellbeing and clinical outcomes among older men with HIV (OMH). Using data from 6 focus groups and 15 interviews with 45 gay, bisexual and heterosexual OMH in Brooklyn, NY and inductive thematic analysis methods, we explored the impact of heteronormative gender roles and ideologies associated with masculinity on healthy aging with HIV. We found that changing physical and sexual function, appearance and growing financial expectations created threats to masculinity, fueling fears of perceived weakness, internalized feelings of shame, depression, anticipated loss of social status (partner loss), and loss of independence and autonomy. These findings suggest that normative gender beliefs, a key social determinant of men’s health, aging and intersectional stigma combined undermine psychosocial support and wellbeing and self-care needed to achieve healthy aging.

